# Diabetes induces remodeling of the left atrial appendage independently of atrial fibrillation in a rodent model of type-2 diabetes

**DOI:** 10.1186/s12933-021-01347-x

**Published:** 2021-07-23

**Authors:** Or Yosefy, Barucha Sharon, Chana Yagil, Mark Shlapoberski, Alejandro Livoff, Ilana Novitski, Ronen Beeri, Yoram Yagil, Chaim Yosefy

**Affiliations:** 1 Department of Cardiology, Barzilai University Medical Center, 2 Hahistadrut Street, 78278 Ashkelon, Israel; 2 Laboratory for Molecular Medicine and Israeli Rat Genome Center, Barzilai University Medical Center, 2 Hahistadrut Street, 78278 Ashkelon, Israel; 3 Department of Pathology, Barzilai University Medical Center, Ashkelon, Israel; 4 Diagnostic Cardiology Unit, Heart Institute, Hadassah Hebrew University Medical Center, Jerusalem, Israel; 5 Faculty of Health Sciences, Ben-Gurion University of the Negev, Beersheba, Israel

**Keywords:** Humans, Heart, Atrial fibrillation, Experimental model, Rats, Cohen diabetic rat, Echocardiography, Glycogen granules

## Abstract

**Background:**

Diabetic patients have an increased predisposition to thromboembolic events, in most cases originating from thrombi in the left atrial appendage (LAA). Remodeling of the LAA, which predisposes to thrombi formation, has been previously described in diabetic patients with atrial fibrillation, but whether remodeling of the LAA occurs in diabetics also in the absence of atrial fibrillation is unknown. To investigate the contribution of diabetes, as opposed to atrial fibrillation, to remodeling of the LAA, we went from humans to the animal model.

**Methods:**

We studied by echocardiography the structure and function of the heart over multiple time points during the evolution of diabetes in the Cohen diabetic sensitive rat (CDs/y) provided diabetogenic diet over a period of 4 months; CDs/y provided regular diet and the Cohen diabetic resistant (CDr/y), which do not develop diabetes, served as controls. All animals were in sinus rhythm throughout the study period.

**Results:**

Compared to controls, CDs/y developed during the evolution of diabetes a greater heart mass, larger left atrial diameter, wider LAA orifice, increased LAA depth, greater end-diastolic and end-systolic diameter, and lower E/A ratio—all indicative of remodeling of the LAA and left atrium (LA), as well as the development of left ventricular diastolic dysfunction. To investigate the pathophysiology involved, we studied the histology of the hearts at the end of the study. We found in diabetic CDs/y, but not in any of the other groups, abundance of glycogen granules in the atrial appendages , atria  and ventricles, which may be of significance as glycogen granules have previously been associated with cell and organ dysfunction in the diabetic heart.

**Conclusions:**

We conclude that our rodent model of diabetes, which was in sinus rhythm, reproduced structural and functional alterations previously observed in hearts of human diabetics with atrial fibrillation. Remodeling of the LAA and of the LA in our model was unrelated to atrial fibrillation and associated with accumulation of glycogen granules. We suggest that myocardial accumulation of glycogen granules is related to the development of diabetes and may play a pathophysiological role in remodeling of the LAA and LA, which predisposes to atrial fibrillation, thromboembolic events and left ventricular diastolic dysfunction in the diabetic heart.

## Background

Diabetic patients have a high prevalence of heart disease [1, 2] and a predisposition to thromboembolic events originating from the heart, leading to stroke and transient ischemic events [3]. Diabetes is also a powerful and independent risk factor for atrial fibrillation [4], the most common predisposing factor to arterial emboli of cardiac origin. The arterial emboli during atrial fibrillation originate in the majority of cases from the LAA.

The LAA, an anatomical structure pouching out of the LA of the heart, is distinct embryonically and anatomically from the atrium [5]. It acts as a “decompression” chamber during left ventricular systole, when atrial pressure is high [6]. The anatomical location and shape of the LAA favor stasis of blood and thrombus formation [7, 8], predisposing to thromboembolic events during atrial fibrillation [9].

In the diabetic patients, what is the contribution of the LAA to the predisposition to thromboembolic events? The high incidence of atrial fibrillation in diabetics appears to be a major inciting factor, and yet additional factors may be involved. Yosefy et al. [10] recently studied a cohort of patients in atrial fibrillation and reported in diabetics, but not in non-diabetics, the occurrence of remodeling of the LAA, which increases the risk for clot formation and thromboembolism. Remodeling of the LAA has previously been attributed to atrial fibrillation [11–13]. Does diabetes by itself predispose to LAA remodeling, unrelated to atrial fibrillation? This question could not be answered in Yosefy’s study [10], as both diabetic and non-diabetic groups were in atrial fibrillation. Other studies focusing on the LAA have also been carried out primarily in patients with atrial fibrillation [14]. Little in fact is known on the effects of diabetes on the structure and function of the LAA.

To determine the contribution of diabetes to LAA remodeling, an important predisposing factor to clot formation and thromboembolism, we studied the heart structure and function in an animal model in sinus rhythm during the evolution of diabetes. We reasoned that if we were to detect LAA remodeling in the animal model during the course of the development of diabetes, such finding would support a causal relationship between remodeling of the appendage and diabetes, dissociate remodeling from atrial fibrillation and establish an experimental platform for investigating the mechanisms involved as well as for testing novel preventive and/or therapeutic measures for the diabetic heart in humans.

Our study hypothesis was that in the diabetic animal with a normal sinus rhythm, structural changes in the LAA occur during the development of diabetes, and that they are causally associated with diabetes and not necessarily related to atrial fibrillation.

## Methods

### Animals

We used the inbred Cohen Diabetic rat, an experimental model of diet-induced type 2 diabetes mellitus, which consists of the sensitive CDs/y and resistant CDr/y strains [15]. When fed RD, both strains maintain a normal metabolic phenotype, but when provided a custom-prepared DD, CDs/y become overtly diabetic within 4 weeks, whereas CDr/y remain non-diabetic [15]. The animals of both strains are normally in sinus rhythm.

We procured the animals from the Israeli Rat Genome Center at the Barzilai University Medical Center in Ashkelon, Israel. Animals were housed up to six in a cage. We maintained 12-h diurnal light-darkness cycles and room temperature at 22–25 °C. We used male animals only. We weaned the animals at age 1 month and provided them with RD and tap water ad libitum.

### Experimental protocol

The use of animals and the experimental protocol were approved by our Institutional Committee for Animal experimentation. The animal procedures were performed in accordance with the NIH guidelines for the care and use of laboratory animals.

#### Animal groups

At baseline, we studied animals fed RD at ~ 6–7 weeks. We then divided the animals into two groups—providing DD or RD. Four groups were thus studied in a 2 × 2 design: The experimental CDs/y-DD group (total at onset n = 18), the diet control CDs/y-RD (total at onset n = 8), CDr/y-DD groups (total at onset n = 9), and the strain CDr/y-RD control group (total at onset n = 7). We used a larger number of animals in the experimental diabetic group in view of anticipated mortality in this group (based on experience from previous studies) and the need to ensure that sufficient animals survive the entire study period.

#### Timeline

We studied the animals at four time points: At “baseline” at age 6–7 weeks; after 1 month of DD or RD, by which time the diabetic phenotype was already fully expressed in CDs/y-DD but not in any other group; after 2.5 months; and after 4 months of DD or RD, to allow the diabetes-related phenotype to evolve in full in the experimental group. We studied both experimental and control strain/diet groups at all time-points, so as to allow us to differentiate between changes attributable to diet, strain or diabetes.

### Echocardiographic studies

We studied the heart structures by echocardiography [16] at baseline and after 1, 2.5 and 4 months of feeding with RD or DD, during which CDs/y-RD, CDr/y-DD and CDr/y-RD remained non-diabetic, whereas CDs/y-DD gradually developed the diabetic phenotype [15, 17]. We sedated the animals with a mixture of xylazine (10 mg/kg)/ketamine (100 mg/kg), 0.15 ml/100 g body weight by intraperitoneal injection, one dose sufficing for the duration of each examination.

The echocardiographic measurements were performed with a conventional GE Vivid 7 echocardiographic machine (GE Vingmed Ultrasound, Horten, Norway), with a linear epi-aortic transducer (L12, GE Vingmed Ultrasound, Horten, Norway). The acquisition included still images and loops in parasternal (long axis and short axis views), apical (4, 5, 2 and 3 chambers) and suprasternal views. An electrocardiogram was recorded to identify end-systole and end-diastole. Imaging parameters were standardized at a frequency 5–10 MHz, depth 2.5 cm, frame rate of at least 125 fps, Doppler sample volume of 1.0 mm and color Doppler aliasing velocity of 40 cm/s. Loops were recorded with at least three heart beats.

We measured or derived the following variables: LA diameter; LAA—orifice length and depth; LVEDD and LVEDD_i_ (corrected per 100 g body mass), LVESD and LVESD_i_ (corrected per 100 g body mass) and LVPWT and LVPWTi (corrected per 100 g body mass); septum—thickness and thickness index; aorta—root diameter and diameter of ascending aorta; and Doppler mitral valve inflow—E and A waves.

### Histopathology

We sacrificed the animals after 2.5 and 4 months of DD or RD (n = 8 in each group). Euthanasia was by exsanguinating the animals from the bifurcation of the aorta under xylazine/ketamine anesthesia, as described above. After exsanguination, we surgically excised the heart, weighed it, fixed it in formaldehyde and embedded it into paraffin blocks. We prepared 4–6 micron sections of the heart, including the ventricles, septum, atria and atrial appendages, and stained the slides with hematoxylin–eosin or PAS without and with diastase. We examined the slides under light microscopy.

### Statistical analysis

We analyzed the data using the Statistica software (TIBCO software Inc., version 15.3). Results are provided as mean ± standard error. Between-group analysis was by one-way ANOVA with the LSD test as the post-hoc analysis measure. We set the significance level at p < 0.05. To test for correlation between variables, we used linear regression analysis.

## Results

### Animal survival

In the experimental CDs/y group provided diabetogenic diet, one animal failed to thrive as it was developing diabetes, as evidenced by failure to increase in weight beyond the lag generally observed in this strain; this animal died between 2.5 and 4 months. Two more CDs/y provided diabetogenic diet and two CDs/y provided regular diet (control group) died during the anesthesia that was necessary for the echocardiographic study. We had previously observed increased sensitivity of the CDs/y strain to anesthesia. As preventive measures, we used as a rule a 10–20% lower dose of anesthetics in this strain and monitored these animals more closely while they were under anesthesia. All CDr/y animals survived the full length of the study.

### Body mass

**Fig. 1:**
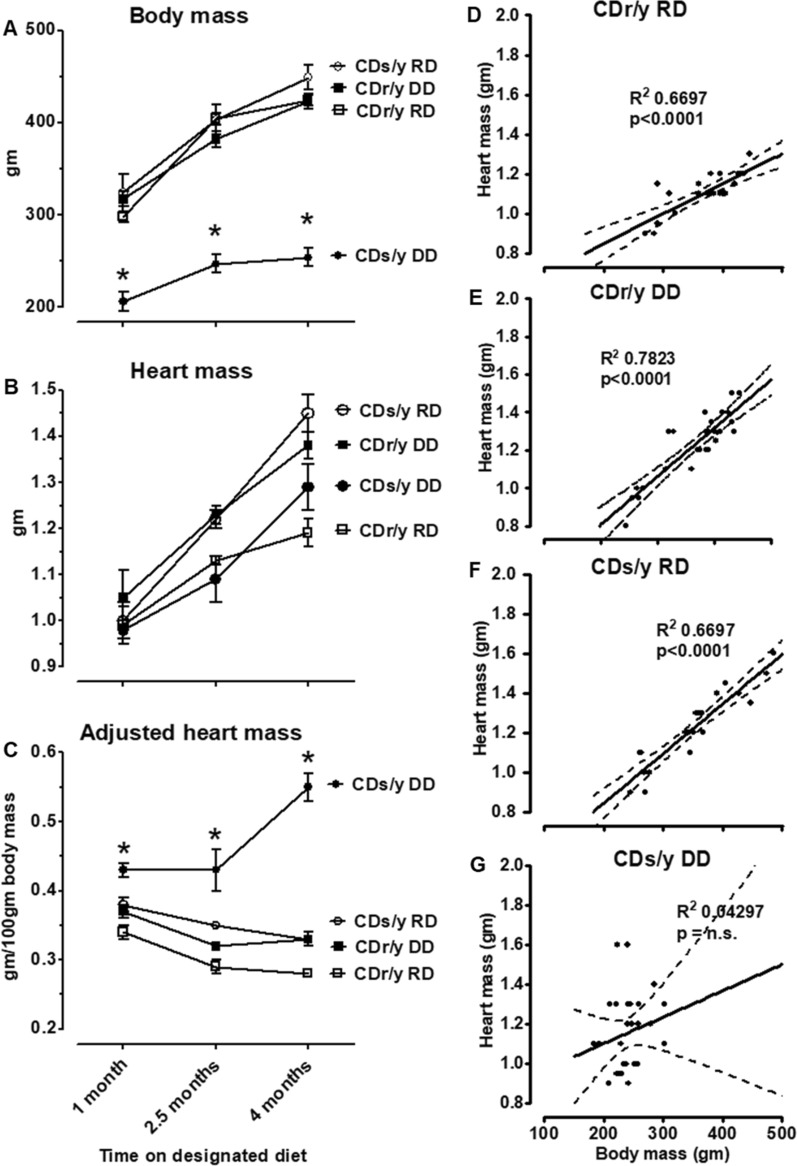
** A** Body mass—CDs/y-DD (n = 15–17), CDs/y-RD (n = 5–7), CDr/y-DD (n = 9) and CDr/y-RD (n = 5–7) after 1, 2.5 and 4 months DD or RD; * p < 0.01 CDs/y-DD compared to all other groups at all time points. **B** Heart mass—CDs/y-DD (n = 7–13), CDs/y-RD (n = 6–10), CDr/y-DD (n = 8–10) and CDr/y-RD (n = 7–10) d after 1, 2.5 and 4 months DD or RD. **C** Heart mass adjusted for body mass—CDs/y-DD (n = 7–13), CDs/y-RD (n = 6–10), CDr/y-DD (n = 8–10) and CDr/y-RD (n = 7–10); after 1, 2.5 and 4 months DD or RD, * p < 0.01 CDs/y-DD compared to all other groups. Correlation of heart with body mass in **D** CDs/y-DD (n = 28), **E** CDs/y-RD (n = 22), **F** CDr/y-DD (n = 27) and **G** CDr/y-RD (n = 24). Statistical analyses: Between group comparison was by one-way ANOVA and LSD, correlation between variables by linear regression with R^2^ and p values

Body mass increased in all groups during the 4 months of the study, but CDs/y-DD lagged behind the other groups (Fig. 1A). As result, the body mass of the diabetic CDs/y-DD group was significantly lower than the three other non-diabetic controls CDr/y-RD, CDr/y-DD and CDs/y-RD at 1 month (F = 29.19, p < 0.001), 2.5 months (F = 57.313, p < 0.001) and 4 months (F = 98.255, p < 0.001).

### Heart mass

The mass of the heart rose significantly and to a similar extent in all groups during the 3 months of the study (Fig. 1B). The increase in heart mass of CDs/y-DD was notable as it occurred despite the sluggish rise in body mass. Consequently, heart mass adjusted for body mass (Fig. 1C) in CDs/y-DD was significantly greater than in the three control groups at 1 month (F = 13.415, p < 0.001), 2.5 months (F = 22.323, p < 0.001) and 4 months (F = 56.919, p < 0.001).

We tested by linear regression if heart mass within each group correlated with body mass, as indication of growth in parallel of heart and body mass (Fig. 1D–G). The correlation was highly significant in CDs/y-RD, CDr/y-DD and CDr/y-RD (p < 0.001). In contrast, the correlation was not significant in CDs/y-DD, indicating that in this group, growth in heart mass was independent of body mass.

### Heart rhythm

All animals were in sinus rhythm at baseline and at 1, 2.5 and 4 months of the study, as demonstrated by echocardiography electrocardiogram (one-lead) and by the presence of E and A waves by doppler echocardiography.

### Echocardiographic studies

#### LA diameter

**Fig. 2:**
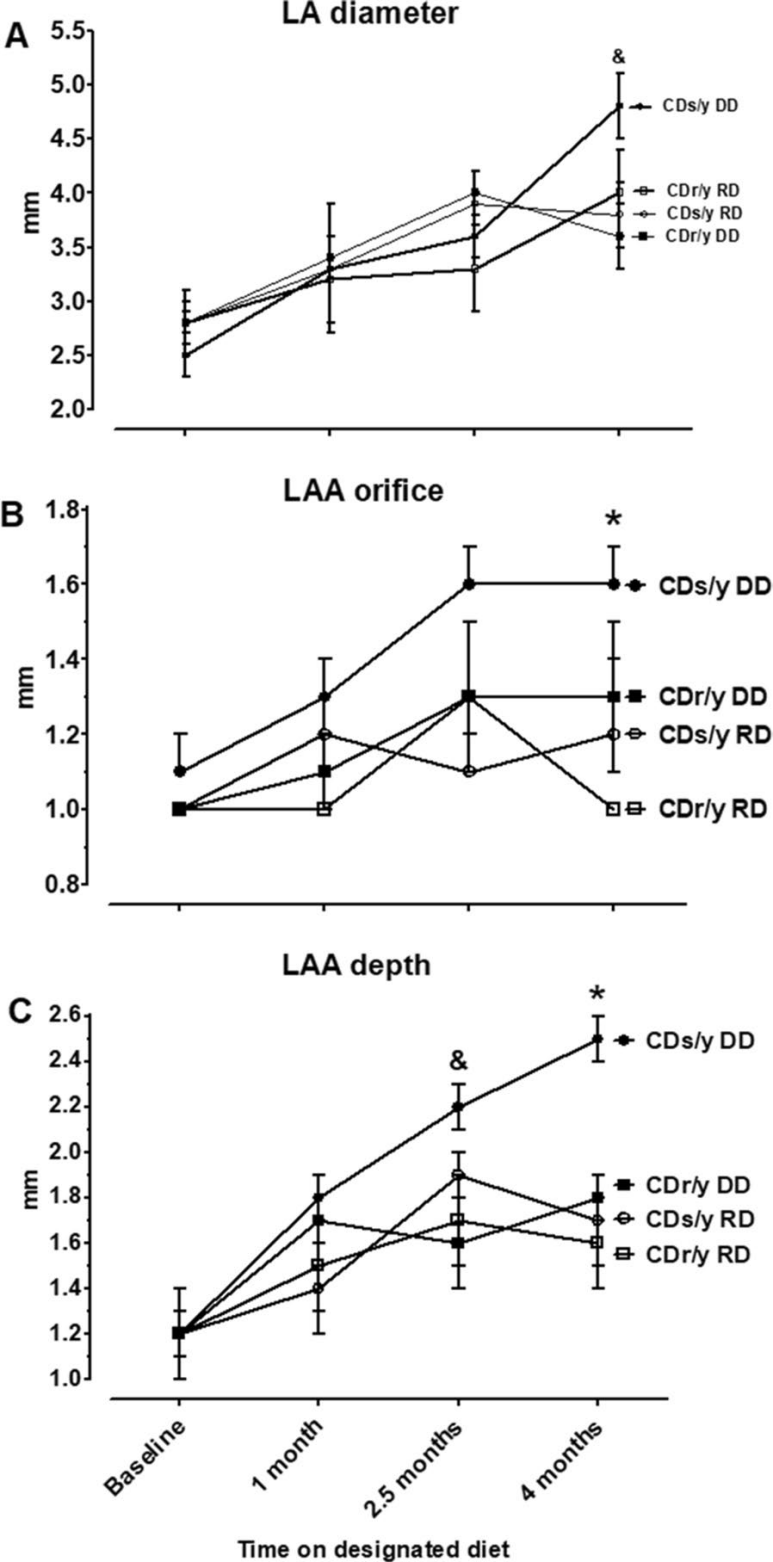
** A** LA diameter—CDs/y-DD (n = 13–17), CDs/y-RD (n = 5–7), CDr/y-DD (n = 8–9) and CDr/y-RD (n = 6–7) at baseline and after 1, 2.5 and 4 months DD or RD; at 4 months—and CDs/y-DD vs CDs/y-RD p = 0.046, vs CDr/y-DD p = 0.009, CDr/y-RD p = 0.082. **B** LAA orifice diameter—CDs/y-DD (n = 13–17), CDs/y-RD (n = 5–7), CDr/y-DD (n = 8–9) and CDr/y-RD (n = 6–7); at 4 months, *CDs/y-DD vs CDs/y-RD p = 0.026, vs CDr/y-DD p = 0.088, CDr/y-RD p = 0.003 and **C** LAA depth—CDs/y-DD (n = 13–17), CDs/y-RD (n = 5–7), CDr/y-DD (n = 8–9) and CDr/y-RD (n = 6–7); at 2.5 months and CDs/y-DD vs CDs/y-RD p = 0.074, vs CDr/y-DD p < 0.001, vs CDr/y-RD p = 0.016; at 4 months *p < 0.001 versus all other groups. Statistical analysis: Between group comparison was by one-way ANOVA and LSD

At baseline and after 1 and 2.5 months of DD or RD, LA diameter was not different among the four study groups (Fig. 2A). At 4 months, however, LA diameter in CDs/y-DD increased and became significantly larger than in all other groups (F 3.168, p = 0.039).

#### LAA

##### Orifice diameter

At baseline and after 1 month, there was no statistically significant difference in the diameter of the orifice to the LAA between the four groups (Fig. 2B). Over the next 3 months, the orifice of the LAA steadily increased in CDs/y and at 4 months tended to be or was significantly larger than in the three other groups (F 0.1907, p = 0.0283).

##### Depth

At baseline, the depth of the LAA was similar in groups (Fig. 2C). As CDs/y-DD became progressively diabetic, the depth of the LAA increased to a greater extent than in all other groups. As result, the depth of the LAA was significantly larger in CDs/y-DD than in the other groups at 2.5 months (F = 5.0879, p = 0.0049) and 4 months (F = 6.7766, p = 0.0012).

#### LV

##### LVEDD

**Fig. 3:**
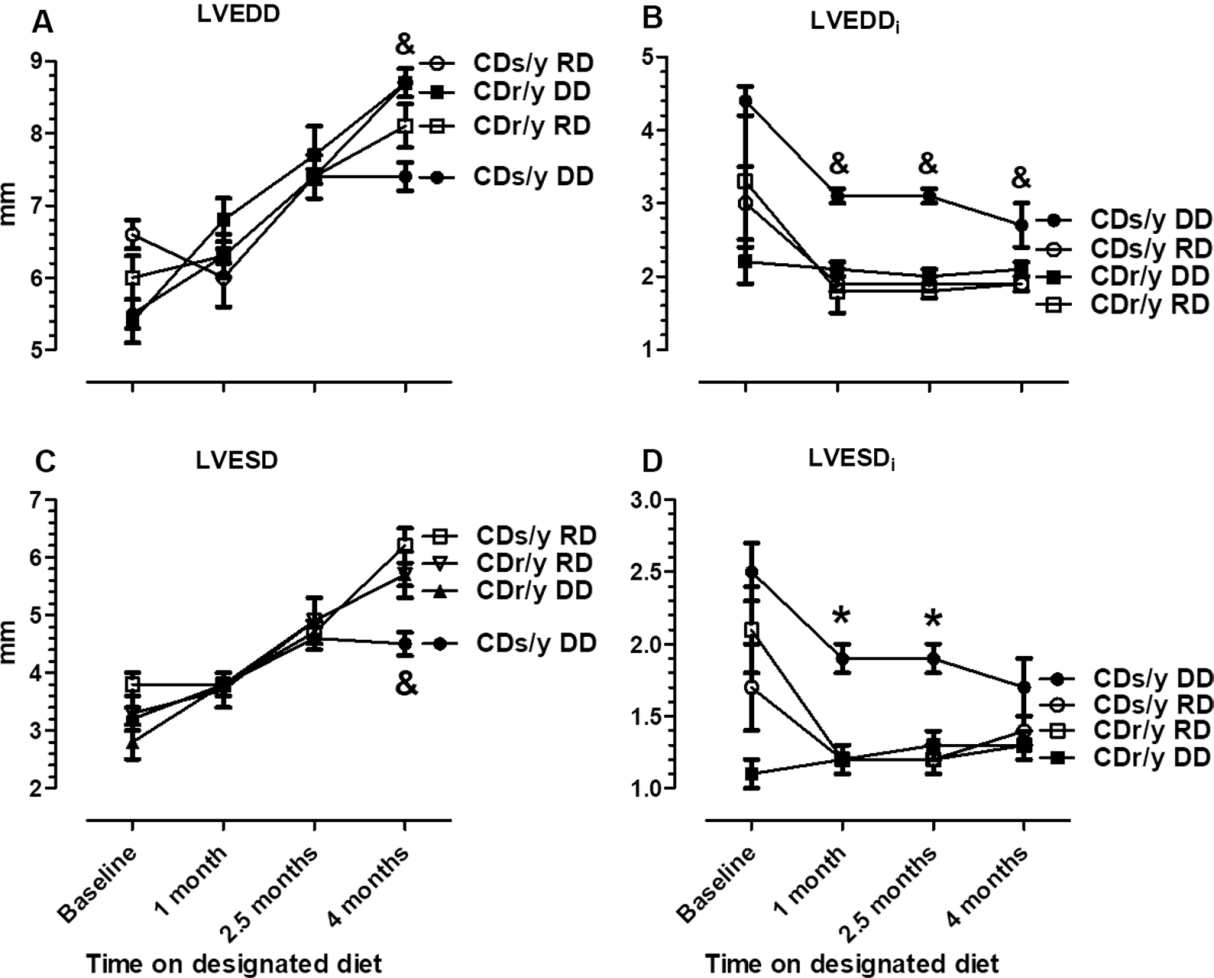
** A** LVEDD—CDs/y-DD (n = 13–17), CDs/y-RD (n = 5–7), CDr/y-DD (n = 9), CDr/y-RD (n = 6–7) at baseline and after 1, 2.5 and 4 months DD or RD; at 4 months—and CDs/y-DD vs CDs/y-RD p < 0.001, vs CDr/y-DD p < 0.001, vs CDr/y-RD p = 0.028. **B** LVEDDi—CDs/y-DD (n = 13–17), CDs/y-RD (n = 5–7), CDr/y-DD (n = 9), CDr/y-RD (n = 6–7); at 1 and 2.5 months—and CDs/y-DD vs CDs/y-RD, vs CDr/y-DD and vs CDr/y-RD p < 0.001, at 4 months—CDs/y-DD vs CDs/y-RD p = 0.016, vs CDr/y-DD p = 0.019 and vs CDr/y-RD p = 0.010; **C** LVESD -CDs/y-DD (n = 13–17), CDs/y-RD (n = 5–7), CDr/y-DD (n = 9), CDr/y-RD (n = 6–7) – at 4 months and CDs/y-DD vs CDs/y-RD and vs CDr/y-DD p < 0.001, vs CDr/y-RD p = 0.006; **D** LVESDi—CDs/y-DD (n = 13–17), CDs/y-RD (n = 5–7), CDr/y-DD (n = 9), CDr/y-RD (n = 6–7); at 1 month and 2.5 months—*CDs/y-DD vs CDs/y-RD, vs CDr/y-DD and vs CDr/y-RD p ≤ 0.001, and at 2.5 months—* CDs/y-DD vs CDs/y-RD, vs CDr/y-DD and vs CDr/y-RD p < 0.001. Statistical analysis: Between group comparison was by one-way ANOVA and LSD

At baseline and until 2.5 months, values were not different between the four groups and increased similarly (Fig. 3A). At 4 months, however, LVEDD in CDs/y-DD was significantly smaller than in all other groups (F = 7.7669, p = 0.0005). In contrast, LVEDDi (Fig. 3B) was significantly higher in CDs/y-DD than in all other groups at 1 month (F = 15.3446, p < 0.001), 2.5 months (F = 27.0840, p < 0.001) and 4 months (F = 4.0287, p = 0.015).

##### LVESD

At baseline and until 2.5 months, there was not difference between the four groups (Fig. 6C). At 4 months LVESD (Fig. 3C) was significantly lower in CDs/y-DD than in all other groups (F = 7.0795, p = 0.0009). LVESD_i_ (Fig. 3D) in contrast, was higher in CDs/y-DD than in all other groups at 1 month (F = 8.9685, p = 0.0002) and 2.5 months (F = 14.8020, p < 0.0000). The differences failed to achieve statistical significance at 4 months, probably due to the relatively large variance in CDs/y-DD.

**Fig. 4:**
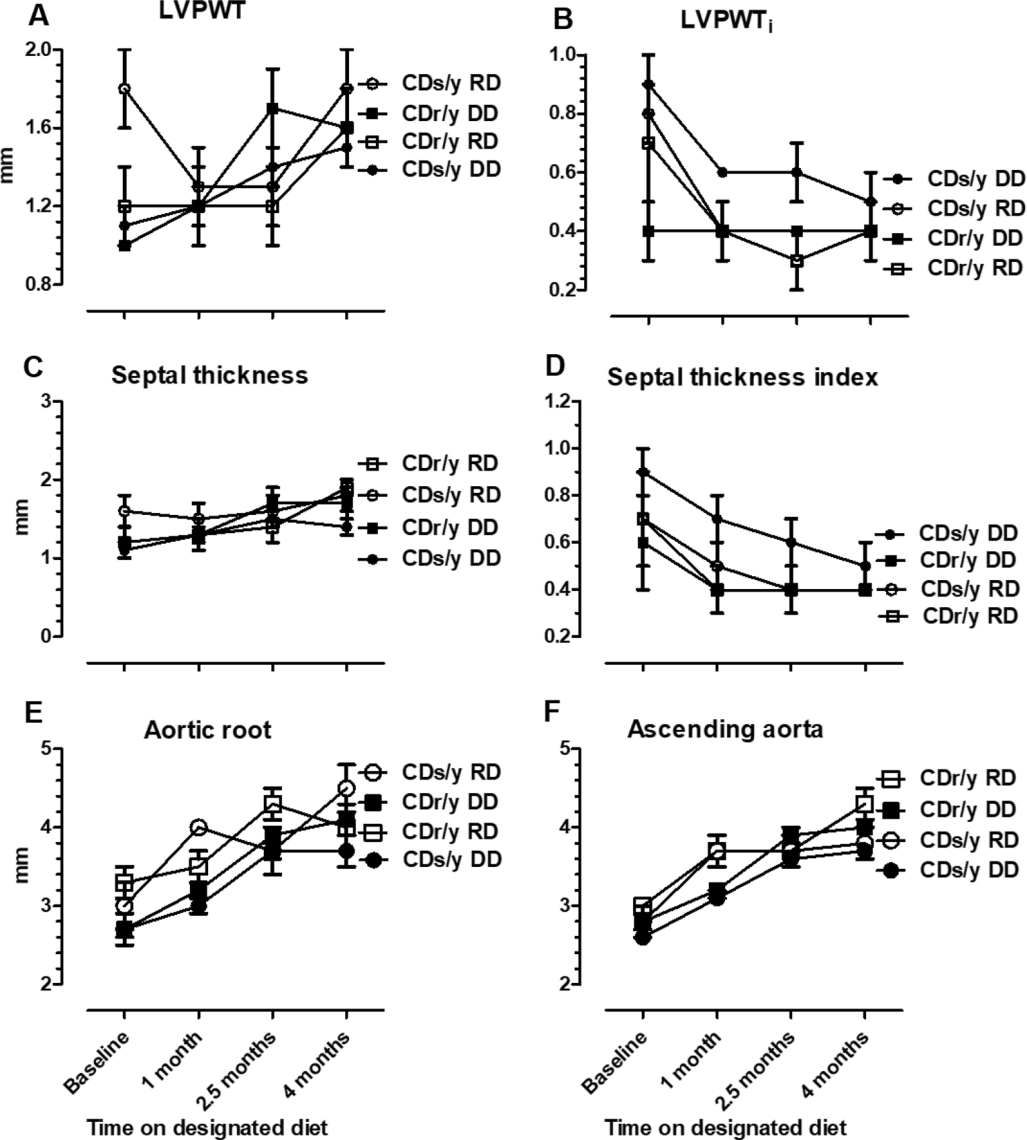
** A** LVPWT—CDs/y-DD (n = 13–17), CDs/y-RD (n = 5–7), CDr/y-DD (n = 9), CDr/y-RD (n = 6–7) at baseline and after 1, 2.5 and 4 months DD or RD. **B** LVPWTi—CDs/y-DD (n = 13–17), CDs/y-RD (n = 5–7), CDr/y-DD (n = 9), CDr/y-RD (n = 6–7). **C** Septal thickness—CDs/y-DD (n = 13–17), CDs/y-RD (n = 5–7), CDr/y-DD (n = 9), CDr/y-RD (n = 6–7). **D** Septal thickness index—CDs/y-DD (n = 13–17), CDs/y-RD (n = 5–7), CDr/y-DD (n = 9), CDr/y-RD (n = 6–7). **E** Diameter of the aorta at the aortic root—CDs/y-DD (n = 12–17), CDs/y-RD (n = 5–7), CDr/y-DD (n = 9), CDr/y-RD (n = 6–7) at baseline and after 1, 2.5 and 4 months DD or RD. **F** Diameter of the aorta at the level of the ascending aorta—CDs/y-DD (n = 12–17), CDs/y-RD (n = 5–7), CDr/y-DD (n = 9), CDr/y-RD (n = 6–7)

*LVPWT* Values were not consistently different between the four study groups, nor was LVPWT (Fig. 4A, B).

###### Septal thickness

Values were not consistently different between the four study groups, nor was there a difference in septal thickness index (Fig. 4C, D).

#### Aorta

There were no consistent differences in the diameter of the aorta at its root nor at the level of the ascending aorta between the four groups at any time point (Fig. 4E, F).

#### Doppler mitral valve flow

##### “E” wave

**Fig. 5:**
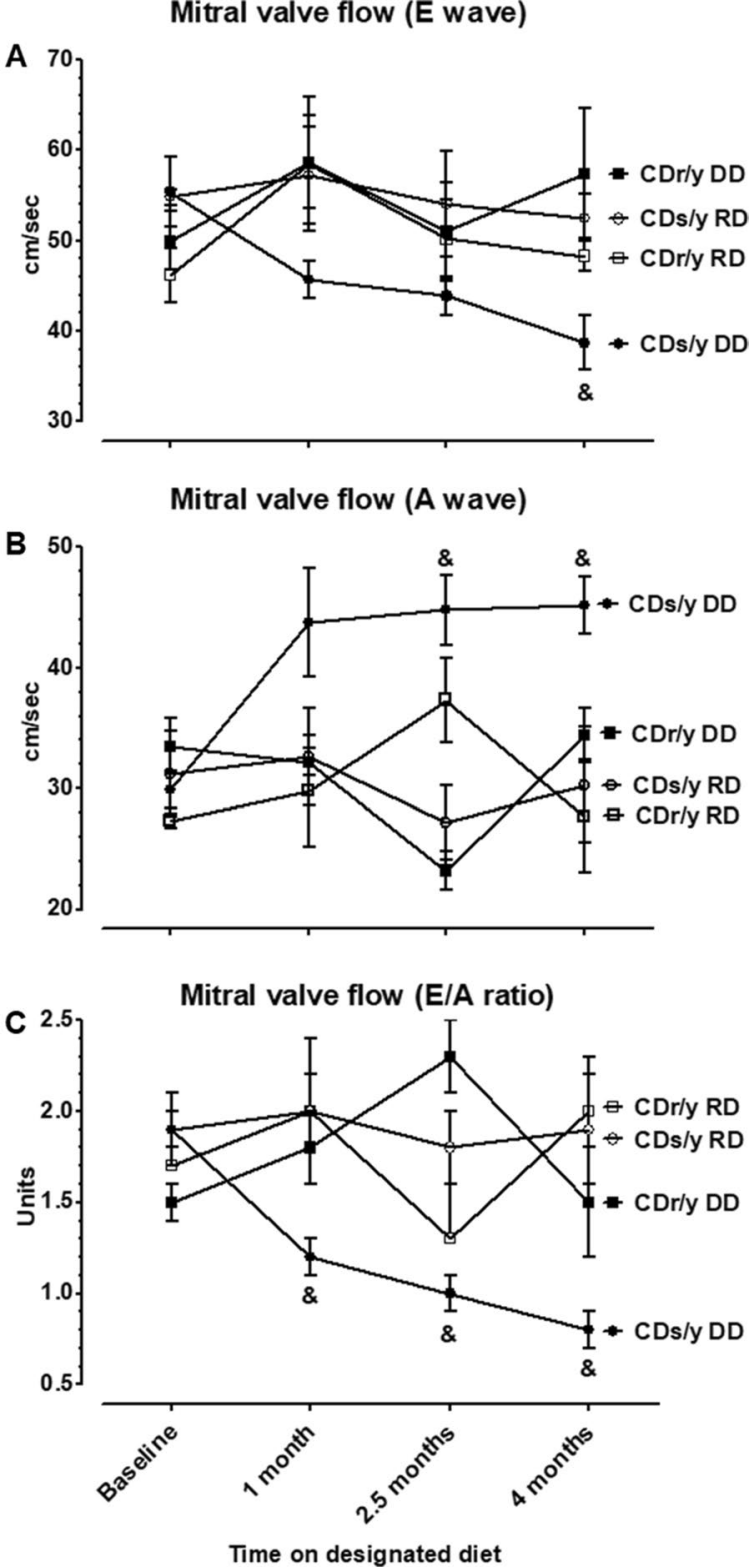
Mitral valve inflow as determined by Doppler echocardiography. **A** E wave—CDs/y-DD (n = 13–17), CDs/y-RD (n = 5–7), CDr/y-DD (n = 8–9), CDr/y-RD (n = 6–7) at baseline and after 1, 2.5 and 4 months DD or RD; at 4 months, and CDs/y-DD vs CDs/y-RD p = 0.031, vs CDr/y-DD p = 0.002, vs CDr/y-RD p = 0.109; **B** A wave—CDs/y-DD (n = 12–15), CDs/y-RD (n = 5–6), CDr/y-DD (n = 8–9), CDr/y-RD (n = 6–7); and at 2.5 months—CDs/y-DD vs CDs/y-RD p = 0.071, vs CDr/y-DD p = 0.003, vs CDr/y-RD p = 0.303 and at 4 months—CDs/y-DD vs CDs/y-RD p = 0.004, vs CDr/y-DD p = 0.017, vs CDr/y-RD p < 0.001; **C** E/A ratio—CDs/y-DD (n = 12–15), CDs/y-RD (n = 5–6), CDr/y-DD (n = 8–9), CDr/y-RD (n = 6–7); at 1 month, and CDs/y-DD vs CDs/y-RD p = 0.013, vs CDr/y-DD p = 0.013, CDr/y-RD p = 0.005, at 2.5 months CDs/y-DD vs CDs/y-RD p = 0.143, vs CDr/y-DD p = 0.001, vs CDr/y-RD p = 0.685 and at 4 months CDs/y-DD vs CDs/y-RD p = 0.003, vs CDr/y-DD p = 0.025, vs CDr/y-RD p < 0.001. Statistical analysis: Between group comparison was by one-way ANOVA and LSD

At baseline, the peak (E) of the pulse wave across the mitral valve (early LV diastolic filling) (Fig. 5A) was not different between the four groups. As of 1 month after initiation of DD, it progressively declined, but in CDs/y-DD to a greater extent than in the other groups, and achieving statistical significance at 4 months (F = 4.223, p = 0.0132).

##### “A” wave

As of 1 month after initiation of DD, the “A” wave (late LV diastolic filling) (Fig. 5B) tended to be higher in CDs/y-DD than in all other groups, achieving statistical significance at 2.5 months (F = 5.9197, p = 0.0025) and 4 months (F = 6.1684, p = 0.0022).

**Fig. 6:**
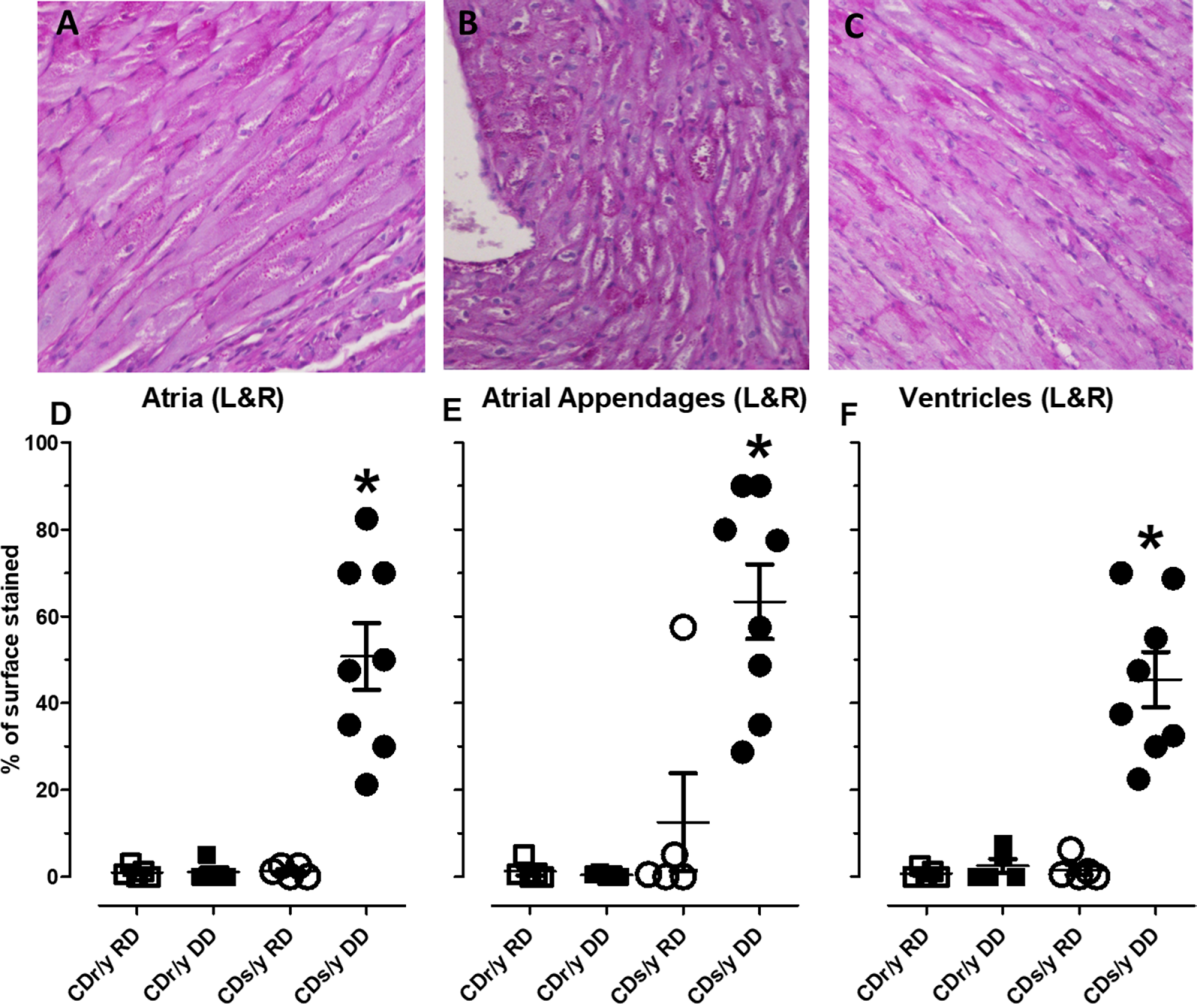
** A**–**C** PAS staining of the representative sections of the atria, atrial appendages and ventricles (×400), respectively, demonstrating abundance of pink staining glycogen granules in and around the myocytes. **D**–**F** showing relative abundance of glycogen granules in the atria, atrial appendages and ventricles of CDr/y-RD (n = 8), CDr/y-DD (n = 8), CDs/y-RD (n = 8) and CDs/y-DD (n = 8); *p < 0.001 CDs/DD vs all other groups. Statistical analysis: Between-group comparison was by one-way ANOVA and LSD

*E/A ratio* As of 1 month after initiation of DD, the E/A ratio (diastolic function of the left ventricle) (Fig. 6C) was significantly reduced in CDs/y-DD compared to all other groups at 1 month (F = 4.7293, p = 0.0083), 2.5 months (F = 5.4427, p = 0.0039) and4 months (F = 6.0857, p = 0.0023).

### Heart histology

We examined histological sections of the heart, including the ventricles, septum, atria and atrial appendages, under light microscopy (×100 and ×400) in the four groups after 2.5 and 4 months of feeding with DD or RD. H&E staining did not reveal differences in the histological appearance of the four groups at both time points. PAS staining revealed at 4 months, but not at 2.5 months, pink-staining granules within the cardiomyocytes in the atria, atrial appendages and ventricles (Fig. 6A–C) of diabetic CDs/y-DD, but not in any of the control groups (not shown). PAS diastase demonstrated that the content of these granules was glycogen (not shown).

To provide a quantitative estimate of the granule content at 4 months, PAS stained slides were blindly examined under light microscopy (×200–400) by two investigators (CY and IN), who estimated independently the percent area covered by glycogen granules in the atria, atrial appendages and ventricles of the four groups. There was a significantly greater amount of glycogen granules in all three heart structures (with no discernable difference between right and left chambers—data not shown) in CDs/y-DD than in all the other groups. Percent surface covered by glycogen granules was 63% in the atrial appendages, 51% in the atria and 45% in the ventricles (Fig. 6D–F), there being no statistically significant difference in glycogen accumulation between the heart chambers in CDs/y-DD (F = 1.4770, p = 0.2511).

## Discussion

In the current study, we focused on the contribution of diabetes to alterations in the heart that predispose diabetic patients to thromboembolic events leading to cerebrovascular events. Our specific target was the LAA, which plays a major role in such events. We went from humans to the animal, seeking to reproduce in a murine model of diabetes mellitus the findings reported by Yosefy et al. [10] in the heart of diabetic patients. We reasoned that such model would lend support to a cause-effect relationship between diabetes and remodeling of the heart in general, and of the LAA in particular.

The phenotype of our model, the Cohen diabetic rat, has been extensively described [15, 17]. One important difference between our model and the human subjects reported by Yosefy et al. [10] is that all the animals in our study were in sinus rhythm, whereas the patients in Yosefy’s study were all in atrial fibrillation. This difference allowed us to differentiate between the role of diabetes mellitus and atrial fibrillation in LAA remodeling.

We began studying our animals after weaning and followed them for 4 months. One finding that immediately stood out was that the growth in body mass of our diabetes prone animal CDs/y-DD during the evolution of diabetes was markedly attenuated compared to the other non-diabetic groups [15, 17]. And yet paradoxically, the heart mass increased steadily in the diabetic CDs/y-DD, similar to the increase observed in the three other control groups. After adjusting for body mass, the increase in heart mass was in fact markedly greater in CDs/y-DD than in all other groups. Interestingly, in the non-diabetic groups, heart mass increased in parallel to the rise in body mass, whereas in diabetic CDs/y-DD, heart mass increased independently of change in body mass. Similar findings have previously been reported by Hsiao et al. [18] in the BB Wistar rat. This disproportionate increase in heart relative to body mass in the diabetic animal raises a question as to the cause for this finding. Hsiao et al. [18] attributed this finding to hypertrophy. Our current study suggests an alternative explanation.

Using echocardiography, we measured the diameter of the orifice to and the depth of the LAA. Initially, both variables did not differ between the groups. After 2.5 and 4 months, both tended to or became significantly greater in the diabetic CDs/y-DD than in the three other non-diabetic groups. These findings suggest remodeling of the LAA in the diabetic animal but not in the other groups, that evolved in parallel to the development of diabetes. Our murine diabetic model thus successfully reproduced the LAA alterations described by Yosefy et al. [10] in diabetic humans with atrial fibrillation. Since remodeling of the LAA occurred solely in the diabetic CDs/y-DD strain, it was unrelated to strain differences (as it did not appear in CDr/y-DD), to diet (as it did not appear in CDs/y-RD) nor to the presence of atrial fibrillation (as all our animals were in sinus rhythm throughout all stages of the study). Therefore, remodeling of the LAA, which promotes blood stasis and clot formation, can be attributed in our model to the development of diabetes mellitus and can be dissociated from atrial fibrillation. This is a central finding in our study, as diabetes-induced remodeling of the LAA, if it also occurs in humans, may play a key role in subsequent embolic complications, accounting for > 90% of transient ischemic attacks or strokes [7].

Previous studies in humans on remodeling of the LAA have been carried out primarily in patients with chronic atrial fibrillation [19, 20]. Epidemiological studies have shown that diabetes mellitus is independently associated with increased risk for atrial fibrillation [21]. What is the link between and sequence of appearance of LAA remodeling, atrial fibrillation and diabetes mellitus? It has been suggested that atrial fibrillation develops first among diabetic patients, leading secondarily to LA [20] and then LAA [14] remodeling.

The current study in our murine model suggests the possibility of a different sequence of events. Our findings indicate gradual enlargement of the LA in the diabetic animal, but only late in the evolution of diabetes, at 4 months of the study. On the other hand, LAA remodeling appears earlier on, after 2.5 months of DD, and consists of structural changes, including a larger orifice and depth leading to lower flow, and functional turning the LAA into a static pouch which predisposes to stagnation, thrombosis and thromboembolism. These findings thus imply that remodeling of the LAA in our murine model precedes that of the LA and is unrelated to atrial fibrillation. Thus, in the presence of sinus rhythm, diabetes may be the primary culprit that leads to LAA remodeling which precedes LA remodeling.

In the course of the study, we detected in CDs/y-DD the development of LV diastolic dysfunction during the evolution of diabetes. The greater LVEDDi and LVESDi in diabetic CDs/y-DD in comparison to the non-diabetic groups as of 1 month of DD suggest primary damage to the LV early in the development of diabetes. The differences in E and A waves and the resulting lower E/A ratio between the diabetic and non-diabetic groups are indicative of diastolic dysfunction during the evolution of diabetes. The lack of consistent increase in septal and posterior wall thickness implies that diastolic dysfunction was due to changes in the myocardial tissue composition of the LV and not due to LV hypertrophy. The lack of change in size of the aorta of CDs/y-DD over the entire study period decreases the likelihood for an aortic contribution to the development of diastolic dysfunction.

In search for an explanation for remodeling of the LAA and the LA, as well as for LV diastolic dysfunction, we studied myocardial histology in the four study groups. Morphological examination revealed a striking finding in diabetic CDs/y-DD, but not in the other groups, consisting of an abundance of glycogen granules in the myocytes of the LAA, atria and ventricles. Glycogen is normally present in most mammalian cells and fulfills a major role in the regulation of cell function, influencing signaling pathways, contractility and gene expression [22]. The extent of glycogen accumulation as granules, consisting of organelle-like “glycosomes” [23], is glucose and time dependent [24, 25]. The association between diabetes and cardiac glycogen accumulation in human subjects is not novel [26] and has also been demonstrated in animal models of diabetes [27–29]. Interestingly, cardiac glycogen accumulation has also been reported in non-diabetic animal models, including after atrial pacing in dogs [30] and prolonged pacing in goats that induced atrial fibrillation and secondary remodeling of the atria [31]. Glycogen accumulation in the latter case has been attributed to stress induced by repeated pacing, an increase in glucose levels and atrial glycogen accumulation [32], leading to alterations in cardiomyocyte function and conduction system, disruption of intracellular communication and increased susceptibility to atrial fibrillation. The association between atrial pacing, diabetes and the accumulation of glycogen granules, has remained, however, inconclusive [33].

The abundance of glycogen granules we found in the experimental CDs/y-DD group but not in the other non-diabetic control groups after 4 months (but not after 2.5 months) of DD stood out as a major pathological finding and may be of pathophysiological significance in relation to cardiac remodeling, as excess glycogen accumulation appears to be detrimental and can lead to cell and organ dysfunction [34]. Our findings lead us to propose that accumulation of glycogen granules may form the basis for the structural and functional changes we observed in the heart of the diabetic animal. We suggest, therefore, that in CDs/y-DD, increased heart/body mass during the evolution of diabetes was due to selective glycogen accumulation in the heart. In the LAA and LA, glycogen granule accumulation during the evolution of diabetes rendered their thin walls increasingly susceptible to atrial and ventricular-induced pressures, thereby allowing stretching of these anatomical structures and resulting in their remodeling. In the LV, glycogen accumulation with the evolution of diabetes may have led to diastolic dysfunction.

Extrapolating our findings to human subjects and returning from the animal model to humans, our starting point in this study, we hypothesize that glycogen accumulation may also occur in the human diabetic heart, accounting at least in part for the development of diabetic cardiomyopathy, with remodeling of the atrial appendages first and subsequently of the atria. The latter would render the diabetic patient susceptible to develop atrial fibrillation and the former to develop thromboembolic complications during atrial fibrillation. Ventricular diastolic dysfunction would be a late sequela. It remains incumbent on us to pursue this hypothesis and demonstrate increased glycogen content in the diabetic heart in humans, a technology that may soon become available [35], and correlate glycogen content with changes in structure and function of the heart during the development of diabetes. If our hypothesis is substantiated, measures would then be focused primarily on improved metabolic control to prevent myocardial accumulation of glycogen granules, which may thus become a primary therapeutic target in the prevention of diabetic heart disease.

## Conclusions

We conclude that our rodent model of diabetes reproduced structural and functional alterations previously observed in hearts of human diabetics with atrial fibrillation. Remodeling of the LAA and of the LA in our murine model was unrelated to atrial fibrillation, as our animals were consistently in sinus rhythm throughout the entire study period, and was associated with accumulation of glycogen granules. Our findings carry an important implication on our understanding of the pathogenesis of the diabetic heart and its complications and suggest that myocardial accumulation of glycogen granules, which is directly related to the development of diabetes, is likely to play a pathophysiological role in remodeling of the LAA and LA, which predispose to atrial fibrillation, thromboembolic events and to left ventricular dysfunction in the diabetic heart. These findings establish our rodent model as an experimental platform for investigating further the mechanisms involved in diabetes-related remodeling of the heart, for continuing research in the prevention of thromboembolic events of cardiac origin in diabetic patients, and for testing of novel preventive and/or therapeutic approaches to the diabetic heart in humans.

## Data Availability

All data generated or analyzed during this study are included in the manuscript.

## Abbreviations

DM: Diabetes mellitus
LAA: Left atrial appendage
CDs/y: Cohen diabetes-prone
CDr/y: Cohen diabetes-resistant
RD: Regular diet
DD: Diabetogenic diet
LA: Left atrium
LV: Left ventricle
LVEDD: Left ventricular end-diastolic diameter
LVEDDi: Left ventricular end-diastolic diameter index
LVESD: Left ventricular end-systolic diameter
LVESDi: Left ventricular end-systolic diameter index
PAS: Periodic acid Schiff
ANOVA: Analysis of variance
LSD: Least significant difference test
LVPWT: Left ventricular posterior wall thickness
LVPWTi: Left ventricular posterior wall thickness index

## References

[1] Kannel WB, McGee DL (1979). Diabetes and cardiovascular risk factors: the Framingham study. Circulation.

[2] Stamler J, Vaccaro O, Neaton JD, Wentworth D (1993). Diabetes, other risk factors, and 12-yr cardiovascular mortality for men screened in the multiple risk factor intervention trial. Diabetes Care.

[3] Mozaffarian D, Benjamin EJ, Go AS, Arnett DK, Blaha MJ, Cushman M (2016). Heart disease and stroke statistics-2016 update: a report from the american heart association. Circulation.

[4] Movahed MR, Hashemzadeh M, Jamal MM (2005). Diabetes mellitus is a strong, independent risk for atrial fibrillation and flutter in addition to other cardiovascular disease. Int J Cardiol.

[5] Leinonen JV, Emanuelov AK, Platt Y, Helman Y, Feinberg Y, Lotan C (2013). Left atrial appendages from adult hearts contain a reservoir of diverse cardiac progenitor cells. PLoS ONE.

[6] Al-Saady NM, Obel OA, Camm AJ (1999). Left atrial appendage: structure, function, and role in thromboembolism. Heart.

[7] Staerk L, Sherer JA, Ko D, Benjamin EJ, Helm RH (2017). Atrial fibrillation: epidemiology, pathophysiology, and clinical outcomes. Circ Res.

[8] Meus R, Son M, Sobczyk D, Undas A (2016). Prothrombotic state in patients with a left atrial appendage thrombus of unknown origin and cerebrovascular events. Stroke.

[9] Menke J, Luthje L, Kastrup A, Larsen J (2010). Thromboembolism in atrial fibrillation. Am J Cardiol.

[10] Yosefy C, Pery M, Nevzorov R, Piltz X, Osherov A, Jafari J (2020). Difference in left atrial appendage remodeling between diabetic and nondiabetic patients with atrial fibrillation. Clin Cardiol.

[11] Kishima H, Mine T, Takahashi S, Ashida K, Ishihara M, Masuyama T (2016). Morphologic remodeling of left atrial appendage in patients with atrial fibrillation. Heart Rhythm.

[12] Shirani J, Alaeddini J (2000). Structural remodeling of the left atrial appendage in patients with chronic non-valvular atrial fibrillation: implications for thrombus formation, systemic embolism, and assessment by transesophageal echocardiography. Cardiovasc Pathol.

[13] Rashid HN, Layland J (2021). Modification of the left atrial appendage and its role in stroke risk reduction with non-valvular atrial fibrillation. Int J Cardiol Heart Vasc.

[14] Yamamoto M, Seo Y, Kawamatsu N, Sato K, Sugano A, Machino-Ohtsuka T (2014). Complex left atrial appendage morphology and left atrial appendage thrombus formation in patients with atrial fibrillation. Circ Cardiovasc Imaging.

[15] Weksler-Zangen S, Yagil C, Zangen DH, Ornoy A, Jacob HJ, Yagil Y (2001). The newly inbred cohen diabetic rat: a nonobese normolipidemic genetic model of diet-induced type 2 diabetes expressing sex differences. Diabetes.

[16] Ribeiro S, Pereira ARS, Pinto AT, Rocha F, Ministro A, Fiuza M (2019). Echocardiographic assessment of cardiac anatomy and function in adult rats. J Vis Exp.

[17] Yagil C, Barkalifa R, Sapojnikov M, Wechsler A, Ben Dor D, Weksler-Zangen S (2007). Metabolic and genomic dissection of diabetes in the Cohen rat. Physiol Genomics.

[18] Hsiao YC, Suzuki K, Abe H, Toyota T (1987). Ultrastructural alterations in cardiac muscle of diabetic BB Wistar rats. Virchows Arch A Pathol Anat Histopathol.

[19] Hensey M, O'Neill L, Mahon C, Keane S, Fabre A, Keane D (2018). A review of the anatomical and histological attributes of the left atrial appendage with descriptive pathological examination of morphology and histology. J Atr Fibrillation.

[20] Litwinowicz R, Bartus M, Ceranowicz P, Brzezinski M, Kapelak B, Lakkireddy D (2019). Left atrial appendage occlusion for stroke prevention in diabetes mellitus patients with atrial fibrillation: long-term results. J Diabetes.

[21] Sun Y, Hu D (2010). The link between diabetes and atrial fibrillation: cause or correlation?. J Cardiovasc Dis Res.

[22] Prats C, Graham TE, Shearer J (2018). The dynamic life of the glycogen granule. J Biol Chem.

[23] Rybicka KK (1996). Glycosomes—the organelles of glycogen metabolism. Tissue Cell.

[24] Brereton MF, Rohm M, Shimomura K, Holland C, Tornovsky-Babeay S, Dadon D (2016). Hyperglycaemia induces metabolic dysfunction and glycogen accumulation in pancreatic beta-cells. Nat Commun.

[25] Andersson LE, Nicholas LM, Filipsson K, Sun J, Medina A, Al-Majdoub M (2016). Glycogen metabolism in the glucose-sensing and supply-driven beta-cell. FEBS Lett.

[26] Mowry RW, Bangle R (1951). Histochemically demonstrable glycogen in the human heart, with special reference to glycogen storage disease and diabetes mellitus. Am J Pathol.

[27] Lajoie C, Beliveau L, Trudeau F, Lavoie N, Massicotte G, Gagnon S (2006). The rapid onset of hyperglycaemia in ZDF rats was associated with a widespread alteration of metabolic proteins implicated in glucose metabolism in the heart. Can J Physiol Pharmacol.

[28] Lajoie C, Calderone A, Trudeau F, Lavoie N, Massicotte G, Gagnon S (2004). Exercise training attenuated the PKB and GSK-3 dephosphorylation in the myocardium of ZDF rats. J Appl Physiol.

[29] Shearer J, Ross KD, Hughey CC, Johnsen VL, Hittel DS, Severson DL (2011). Exercise training does not correct abnormal cardiac glycogen accumulation in the db/db mouse model of type 2 diabetes. Am J Physiol Endocrinol Metab.

[30] Zhang L, Ji XP, Zhang W, Wang R, Jiang SL, Chen WQ (2007). Atrial electrical, contractile and structural remodeling induced by short-term atrial tachycardia in a canine model. Zhonghua Xin Xue Guan Bing Za Zhi.

[31] Embi AA, Scherlag BJ, Ritchey JW (2014). Glycogen and the propensity for atrial fibrillation: intrinsic anatomic differences in glycogen in the left and right atria in the goat heart. N Am J Med Sci.

[32] Embi AA, Scherlag BJ (2014). An endocrine hypothesis for the genesis of atrial fibrillation: the hypothalamic-pituitary-adrenal axis response to stress and glycogen accumulation in atrial tissues. N Am J Med Sci.

[33] Maria Z, Campolo AR, Scherlag BJ, Ritchey JW, Lacombe VA (2018). Dysregulation of insulin-sensitive glucose transporters during insulin resistance-induced atrial fibrillation. Biochim Biophys Acta Mol Basis Dis.

[34] Ashcroft FM, Rohm M, Clark A, Brereton MF (2017). Is type 2 diabetes a glycogen storage disease of pancreatic beta cells?. Cell Metab.

[35] Zhou Y, van Zijl PCM, Xu X, Xu J, Li Y, Chen L (2020). Magnetic resonance imaging of glycogen using its magnetic coupling with water. Proc Natl Acad Sci USA.

